# Crosstalk of Epigenetic and Metabolic Signaling Underpinning Glioblastoma Pathogenesis

**DOI:** 10.3390/cancers14112655

**Published:** 2022-05-27

**Authors:** Mariam Markouli, Dimitrios Strepkos, Kostas A. Papavassiliou, Athanasios G. Papavassiliou, Christina Piperi

**Affiliations:** Department of Biological Chemistry, Medical School, National and Kapodistrian University of Athens, 11527 Athens, Greece; smd1800087@uoa.gr (M.M.); smd1700150@uoa.gr (D.S.); konpapav@med.uoa.gr (K.A.P.)

**Keywords:** glioma, glioblastoma, methylation, acetylation, histones, DNA, glycolysis, TCA cycle, Krebs cycle, gluconeogenesis, oxidative phosphorylation, pentose phosphate pathway, microRNAs, glutamine

## Abstract

**Simple Summary:**

Epigenetic mechanisms can modulate key genes involved in the cellular metabolism of glioblastomas and participate in their pathogenesis by increasing their heterogeneity, plasticity, and malignancy. Although most epigenetic modifications can primarily promote the activity of metabolic pathways, they may also exert an inhibitory role. The detection of key metabolic alterations in gliomas regulated by epigenetic mechanisms will enable drug development and effective molecular targeting, improvement of therapeutic schemes, and patients’ management.

**Abstract:**

Metabolic alterations in neoplastic cells have recently gained increasing attention as a main topic of research, playing a crucial regulatory role in the development and progression of tumors. The interplay between epigenetic modifications and metabolic pathways in glioblastoma cells has emerged as a key pathogenic area with great potential for targeted therapy. Epigenetic mechanisms have been demonstrated to affect main metabolic pathways, such as glycolysis, pentose phosphate pathway, gluconeogenesis, oxidative phosphorylation, TCA cycle, lipid, and glutamine metabolism by modifying key regulatory genes. Although epigenetic modifications can primarily promote the activity of metabolic pathways, they may also exert an inhibitory role. In this way, they participate in a complex network of interactions that regulate the metabolic behavior of malignant cells, increasing their heterogeneity and plasticity. Herein, we discuss the main epigenetic mechanisms that regulate the metabolic pathways in glioblastoma cells and highlight their targeting potential against tumor progression.

## 1. Introduction

Gliomas, and glioneuronal and neuronal tumors comprise the majority of primary central nervous system (CNS) neoplasms [1]. The latest 2021 edition of the WHO Classification for Central Nervous System Tumors differentiates these tumors into six different families. The first one comprises the adult-type diffuse gliomas, which constitute most of the adult primary brain tumors, including isocitrate dehydrogenase (IDH)-wild type (WT) glioblastoma (GB). The other five families include pediatric low- and high-grade gliomas (a subcategory of which are the diffuse midline gliomas with histone 3 lysine 27 replaced by a methionine (H3K27M) mutation), circumscribed astrocytic gliomas, glioneuronal and neuronal tumors, and ependymomas [2].

Astrocytic tumors are further sub-grouped into different grades with the use of Arabic numbers [2], depending on their histological type. The new grading system was organized so that each disease entity can only receive a predetermined number of grades. For example, IDH-mutant astrocytomas can be graded as 2, 3, or 4 (replacing the old term “IDH-mutant GB”), while an IDH-wild type GB, of the adult-type diffuse glioma family can only be grade 4. GB is the most common primary brain tumor, accounting for almost half of the total malignant primary CNS tumors [3]. Unfortunately, it is associated with an abysmal prognosis, with only 5.5% of patients surviving past the 5 years after diagnosis [4] and the median patient survival being as low as 15 months [5].

Glioblastoma diagnosis relies on the presence of isocitrate dehydrogenase (IDH) mutations, a key enzyme of the tricarboxylic acid (TCA or Krebs) cycle. The formerly named IDH-mutated GBs (now termed IDH-mutant astrocytomas grade 4) and IDH-wild type GBs represent two distinct, large categories of tumors. IDH-wild type GB that accounts for approximately 90% of GB cases is considered a primary tumor and is associated with mutations at the telomerase reverse transcriptase (TERT) promoter, amplification of the epidermal growth factor receptor (EGFR), and +7/−10 copy number changes [2,6]. Conversely, IDH-mutated (mIDH) grade 4 astrocytomas are considered secondary, arising from previous lower grade diffuse gliomas [7], and they have been associated with homozygous deletion of *cyclin-dependent kinase 2A/B* (CDKN2A/B) [2].

The GB epigenome is currently under extensive investigation in order to explore the respective post-translational modifications that drive the development and progression of these tumors. Epigenetic alterations constitute a range of chemical modifications that affect gene expression and function without altering nucleotide sequence [8]. They include DNA modifications, such as DNA methylation; acetylation; ubiquitinylation; demethylation; as well as non-histone protein and histone post-translational changes, such as methylation, acetylation, ubiquitinylation, and demethylation, occurring primarily at the N-terminals. Additionally, non-coding RNAs (ncRNAs) can interfere with and modify the expression of genes in an epigenetic manner. These RNAs include small interfering RNA (siRNAs), microRNAs (miRNAs), long noncoding RNAs (lncRNAs), small nuclear RNAs (snRNA), and small nucleolar RNAs (snoRNAs).

DNA methylation, catalyzed by DNA methyltransferases (DNMTs), involves the addition of methyl groups supplied by S-adenosyl methionine (SAM) to form 5-methylcytosine in CpG dinucleotides [9], which leads to transcriptional gene silencing. On the other hand, histone methylation is carried out by a variety of histone methyltransferases (HMTs), depositing methyl marks on specific histones, and inducing rearrangement of chromatin structure. A shift from a less condensed and “relaxed” chromatin state to a more condensed and “closed” state takes place [10], which inhibits transcriptional factor and enzyme accessibility to gene promoters. Histone methylases are able to methylate lysine, arginine, and histidine residues at the N-terminal tails and globular domains of histones [11]. The lysine histone methylation is carried out by the SET domain-containing and the Dot1-like families of proteins [12,13] while arginine methylation is carried out by the protein arginine methyltransferase (PRMT) family of proteins [11]. Both types of lysine and arginine methylation may also occur on non-histone proteins [14], a post-translational modification that interferes with histone methylation and can regulate many cellular processes [15]. Contrary to histone methylases, histone acetylases work to promote gene expression, making the chromatin structure more accessible to transcription factors and enzymes [16]. Regarding miRNAs, they are able to associate with complementary sequences, forming complexes and reducing the available levels of specific RNAs while decreasing the production of the final protein products [17]. Lastly, non-histone protein post-translational modifications are crucial in the regulation of gene expression [18]. Methylation of these proteins can occur in either lysine or arginine residues and is carried out by protein lysine and protein arginine methyltransferases (PKMTs and PRMTs) [19]. Furthermore, demethylation as well as acetylation and deacetylation are other examples of post-translational modifications of non-histone proteins that ultimately affect gene expression. All of the above-mentioned epigenetic alterations provide a wide range of tools employed by the cell to reversibly control the expression of key regulatory genes, including those that control its metabolism.

A common finding in GBs is the alterations in cellular metabolism in comparison with physiologic glial cells. A great example includes the mutations of metabolic enzymes such as IDH, which are used to differentiate between IDH-wild type GB and IDH-mutant grade 4 astrocytomas. Importantly, one of the most well-documented metabolic alterations in cancer is the Warburg effect, which refers to the switch from oxidative phosphorylation to aerobic glycolysis in the presence of oxygen and functioning mitochondria [20]. Cancer cells utilize glucose in a significantly less efficient manner, producing less ATP, despite being able to catabolize glucose through the regular oxidative phosphorylation pathway. Consequently, glucose uptake takes place by respiring cells, which undergo glycolysis, generating pyruvate as end-product and entering the TCA cycle, thus producing reduced NADH. The latter is then used by the electron transport chain (ETC), generating 36 ATP molecules for each glucose molecule. On the other hand, during aerobic glycolysis, pyruvate is converted into lactate, yielding only two molecules of ATP [21]. This switch, however, exhibits various beneficial effects on constantly multiplying cancer cells in order to cover the need for a rapidly available energy source, since lactate production from glucose takes place 10–100 times faster than the complete mitochondrial glucose oxidation [20]. Therefore, cancer cells deploy the Warburg effect to secure their energy requirements, even though it is not as efficient in producing ATP as oxidative phosphorylation [22]. Glycolysis and the pentose phosphate pathway also meet the biosynthetic needs of cancer cells [23,24] and help to acidify the tumor microenvironment through lactate production, which promotes tumor invasion [25]. Lastly, oxidative phosphorylation presents a source of harmful reactive oxygen species (ROS), which cause severe damage to cancer cells. By shutting down the glycolytic pathway, cancer cells create a favorable environment for tumor growth [8]. Notably, hypoxia, which leads to accumulation of hypoxia inducible factor (HIF) can promote glycolysis at the expense of oxidative phosphorylation [9].

A therapeutic problem which has arisen with the identification of this “modified” tumor metabolism is the different cell populations present in GB tumors, with some cells comprising the main part of the tumor and others serving the tumor regenerative purposes, such as glioma stem cells (GCSs) [10]. Studies have unveiled that each malignant cell subtype displays different uses of metabolic enzymes and exploits different pathways to serve its energy needs [16]. GSCs for example exhibit lower glycolytic activity when compared with their differentiated progeny and therefore consume less glucose, produce less lactate, and maintain higher ATP levels. It is therefore evident that GSCs rely on oxidative metabolism and that they are not affected by any attempts to downregulate glycolysis [16]. This potential increases the heterogeneity of the different GB subtypes, allowing for the presence of cells with unique gene expression programs within each tumor [17]. Recently, studies have shown that GB cells can change the expression of their metabolic enzymes based on the stimuli received from the microenvironment, such as the available nutrients [26]. This ability of GB cells contributes to their plasticity and allows them to adapt in order to survive.

It is important to note that metabolic changes not only occur in cancer cells themselves but also characterize cells participating in the formation of the tumor microenvironment. Most importantly, changes in immune cell metabolism associated with epigenetic modifications that influence gene expression appear to drastically affect immune cell function and contribute to tumorigenesis [27]. Examples include several metabolic changes aiming to meet the increasing energy demands of trained macrophages, such as the shift to aerobic glycolysis from oxidative phosphorylation, which correlate with DNA methylation, as well as histone modifications [27,28].

It therefore becomes evident that alterations in malignant cell metabolism are of major importance for glioblastoma progression, stemness, heterogeneity, and plasticity. To investigate the regulation of all these metabolic changes, epigenetic mechanisms have emerged as main contributors of these alterations. The interaction between epigenetic changes and metabolic pathways presents a very promising way of targeting malignant cells, while elucidation of the metabolic pathways exploited by cancer cells for their progression provides a new array of therapeutic targets.

In this review, we discuss current experimental data on the diverse metabolic changes taking place in GB cells and their regulation by epigenetic modifications. We further discuss how the interplay of epigenetic mechanisms and cell metabolism can be targeted to improve GB management.

## 2. Metabolic Pathways Regulated by Epigenetic Mechanisms in Glioblastomas

Although GB cells rely mostly on aerobic glycolysis, as pointed out by the Warburg Effect, additional metabolic pathways can be employed by neoplastic cells, which can undergo a series of alterations that are often dictated by epigenetic modifications.

### 2.1. Epigenetic Regulation of Glycolysis

#### 2.1.1. Effects of DNA Methylation in Glycolysis

Glycolysis in GB is affected by DNA methylation, mainly promoting gene silencing. Experimental evidence indicates the upregulation of glucose transporter (GLUT), which enhances the cellular uptake of glucose, thus promoting aerobic glycolysis. Specifically, promoter CpG island (CGI) hypermethylation inactivates derlin-3 (DERL3), which is responsible for degrading GLUT1 and therefore increasing GLUT expression and aerobic glycolysis [29]. On the other hand, CpG promoter site hypomethylation causes the abnormal elevation of caveolin-1 (CAV-1), which then stimulates GLUT3 transcription, again favoring glucose uptake and aerobic glycolysis [30]. Additionally, the expression of glycolytic enzymes can be regulated by DNA methylation. Pyruvate kinase (PK) isoenzyme M2, the M2 isoform of PK that favors aerobic glycolysis by catalyzing its rate-limiting step, namely the conversion of phosphoenolpyruvate into pyruvate, depends on the intron 1 hypomethylation status of its respective gene, pyruvate kinase M1/2 (*PKM*) [31]. Intragenic methylation has been shown to promote the attachment of brother of regulator of imprinted sites (BORIS) on one of its exons, causing an alternative splicing that enhances the Warburg effect. When DNA methylation or the BORIS attachment site are lost, splicing may take place, which generates the PKM1 isoform [32]. Methylation of PKM2 by the coactivator-associated arginine methyltransferase 1 (CARM1) at three of its arginine residues (R445, R447, and R455) localizes the enzyme to the mitochondrial endoplasmic reticulum (ER) membrane. This decreases the membrane potential and Ca^2+^ uptake, which are essential events for the activation of pyruvate dehydrogenase (PDH) in favor of oxidative phosphorylation (OXPHOS), thus aiding aerobic glycolysis [9]. Moreover, the hypomethylation of the hexokinase 2 (HK2) promoter, an enzyme that catalyzes the first step of glycolysis, leads to HK2 upregulation in GB [33], increasing aerobic glycolysis [34].

**Figure 1:**
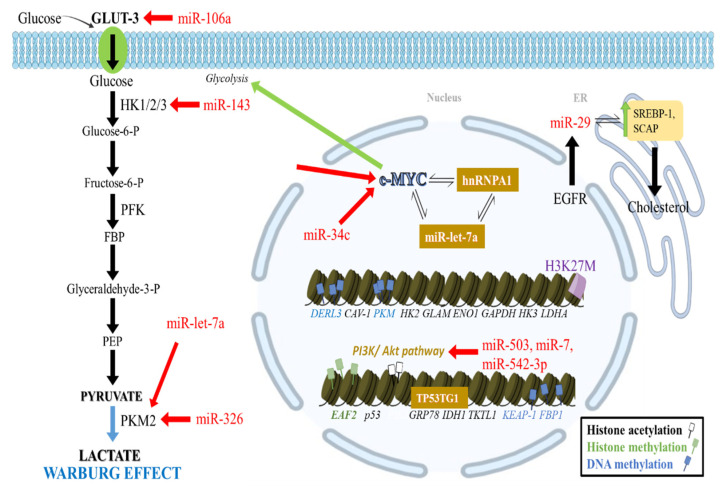
Crosstalk of epigenetic changes with glycolysis and lipid metabolism in GB. CpG promoter hypomethylation increases CAV-1 expression, stimulating GLUT3 transcription. Moreover, *HK2* promoter hypomethylation and *PKM* hypomethylation promote aerobic glycolysis. Additional glycolytic gene promoters in mesenchymal subtype GBs, including *ENO1*, *GLAM*, *HK3*, *GAPDH*, and *LDHA*, are further upregulated by hypomethylation. Elevated histone mark H3K27me3 expression on the *EAF2* promoter favor its transcription and a shift towards glycolysis. Histone deacetylation has been involved in the elevation of c-myc expression, which interacts with miR-let-7a, PKM2, and hnRNPA1 to ensure PKM2 upregulation. MiR-let-7a and miR-34c inhibit PKM2 and c-myc signaling. TP53TG1 lncRNA increases *GRP78* and *IDH1* expression. MiRNAs can downregulate GLUT3 and inhibit glycolysis at various levels (HK1/2/3 and PKM2) as well as the PI3K/Akt pathway. Diffuse midline gliomas with H3K27M mutation, exhibit upregulation of glycolysis and the TCA cycle. In addition, OXPHOS is increased, and lipid metabolism is shifted towards cholesterol synthesis. This is achieved by a network of molecules including EGFR, which upregulates miR-29 that then increases SREBP-1 and SCAP.

Regarding IDH-mutated grade 4 astrocytomas, the promoter areas of several glycolytic enzyme genes, such as *GLAM*, enolase 1 (*ENO1*), glyceraldehyde-3-phosphate dehydrogenase (*GAPDH*), hexokinase 3 (*HK3*), and lactate dehydrogenase A (*LDHA*), are hypermethylated [35], leading to decreased gene expression and enzyme production. On the contrary, the mesenchymal subtype GBs are characterized by promoter region hypomethylation and thus increased glycolytic enzyme gene expression [35] (Figure 1).

#### 2.1.2. Effects of Histone Methyltransferases and Demethylases in Glycolysis

Histone methyltransferase activity and respective histone marks have been shown to affect glycolysis in GB [31]. In particular, the G9a methyltransferase has been found downregulated during hypoxic states in GBs. On the other hand, the enhancer of zeste 2 polycomb repressive complex 2 subunit (EZH2) methyltransferase increases H3K27me3 levels on the ELL associated factor 2 (*EAF2*) promoter, favoring *EAF2* transcription and promoting HIF1α-mediated signaling, thus enabling the shift from mitochondrial respiration to glycolysis in GB [36] (Figure 1). In addition, the methyltransferases G9a and G9a-like protein (GLP) have been shown to bind directly to HIF-1α and to cause K674 mono- and dimethylation, which then represses HIF-1 transcriptional activity. HIF-1 repression further decreases downstream target genes expression, including prostaglandin-endoperoxide synthase 1 (*PTGS1*), solute carrier family 6 member 3 (*SLC6A3*), long intergenic non-protein coding RNA 1132 (*Linc01132*), and neuron-derived neurotrophic factor (*NDNF*) [37]. Another histone methyltransferase, mixed lineage leukemia 1 (MLL1) was also shown to inhibit the HIF transcript, HIF2α protein, and the expression of target genes. Some of these target genes were demonstrated to code for glycolytic enzymes [38], such as Gluts, HKs, LHDA, and phosphofructokinase (PFK) [39]. Regarding arginine methylation, studies have shown that PRMT5 is involved in the dysregulation of metabolism in cancer cells. PRMT5 plays a crucial role in the efficacy of the Warburg effect. When the cancer cell has an abundance of intracellular glucose, PRMT5 works to promote its transition from the G1 to the S phase by upregulating CDK4 [40]. In more detail, PRMT5 is responsible for the symmetrical methylation of histone 3 arginine 2 (H3R2) residue in the promoter of genes involved in gluconeogenesis, leading to increased hepatic glucose production [41].

Histone demethylases (HDMs) may also influence the glycolytic pathway in GB. Histone lysine demethylase 1 (LSD1 or HDM1A) suppresses the p53 pathway, a regulator of glucose metabolism, leading to the inhibition of GSC terminal differentiation [31]. In addition, the miR-215/lysine demethylase 1B (KDM1B) axis was shown to control the hypoxic response and expression of genes involved in glucose metabolism including *HIF2α,* N-Myc downstream regulated 1 (*Ndrg1*), adrenomedullin (*ADM*), NDUFA4 mitochondrial complex associated like 2 (*NDUFA4L2*), *Glut1*, and *Glut3* in glioma-developing cells. In more detail, miR-215 suppresses KDM1B, resulting in the activation of the anaerobic switch that promotes glycolysis [42].

#### 2.1.3. Effects of Histone Acetyltransferases and Deacetylases in Glycolysis

Histone acetylation favors gene expression and has been observed to affect glycolysis in GB. The histone acetyltransferase PCAF (KAT2B) acetylates AKT serine/threonine kinase 1 (Akt1) and promotes its phosphorylation at T308 and S473, which ultimately results in GB cell proliferation and increased glycolysis [43]. Similarly, the histone lysine acetyltransferase 6A (KAT6A) catalyzes H3K23 acetylation and recruits the nuclear receptor binding protein tripartite motif containing 24 (TRIM24) in order to activate phosphoinositide-3-kinase, catalytic, alpha polypeptide (*PIK3CA*) transcription, which further promotes Akt phosphorylation [44]. The phosphatidylinositol 3-kinase (PI3K)/Akt signaling pathway then promotes glycolytic gene expression, such as *Glut1*, *PFK1*, and *HK1/2/3* [45].

Histone deacetylation is also involved in the regulation of glycolysis in GB. The glycolytic regulator mechanistic target of rapamycin complex 2 (mTORC2) phosphorylates, and destabilizes or decreases the expression of histone deacetylase (*HDAC*) 4, 5, and 7. This inactivation results in forkhead Box (Fox) O1/O3 acetylation, which in turn decreases miR-34c transcription and increases c-Myc expression, favoring the glycolytic metabolism of GB [46] by inducing the expression of the glycolytic genes *Glut1*, *LDH-A, ENO1*, and serine hydroxymethyl transferase (*SHMT*) [47]. On the other hand, Sirtuin 6 (SIRT6) is a HDAC that recruits the myeloid zinc finger 1 (MZF1) transcription factor and acts as its co-repressor by forming the SIRT6–MZF1 complex, in turn preventing *HK2* transcription and inhibiting glycolysis [48].

In regard to immune cells, aerobic glycolysis is a hallmark of the metabolic properties of activated T-cells and helps enhance effector T-cell activity. In more detail, lactate dehydrogenase A is enhanced in activated T-cells in order to sustain increased aerobic glycolysis levels, enhancing the proinflammatory cytokine IFN-γ expression. This enzyme aids in maintaining high acetyl-CoA levels, promoting histone acetylation and *Ifng* transcription, and possibly playing a role in immune responses in cancer [49,50].

#### 2.1.4. Noncoding RNA Effects in Glycolysis

Circular RNAs (circRNAs) have been associated with the pathogenesis of various tumors. In more detail, circPITX1 and NEK2 appear to be upregulated in gliomas and circPITX1 knockdown resulted in decreased glycolysis, viability, and cancer colony formation. Its knockdown also promoted glioma cell radiosensitivity and inhibition of tumor growth in vivo [51].

Concerning long-coding RNAs, the lncRNA LEF1 Antisense RNA 1 (LEF1-AS1) was demonstrated to promote GB proliferation as well as invasion, while also inhibiting apoptosis through the signaling pathway of Akt/mTOR, which is involved in regulation of glycolysis [52].

Additionally, GLUT3 can be targeted by the microRNA miR-106a to reduce glucose flux [53], whereas miR-143 targets HK2 to block glycolysis and enhance differentiation of GB stem-like cells [54]. MiR-326 as well as miR-let-7a repress PKM2 expression [55,56]. Moreover, miR-7, miR-128, and miR-219-5p have been found downregulated in GB. They normally inhibit EGFR expression, which aids in protein kinase C epsilon (PKCε) monoubiquitylation, activates nuclear factor kappa beta (NF-κB), and upregulates *PKM2*, subsequently promoting glycolysis and tumorigenesis [47,57,58,59,60,61]. On the contrary, miR-21 targets the transcriptional activator of *EGFR*, signal transducer and activator of transcription 3 (STAT3) and is positively associated with increased malignancy grade and reduced patient survival [62]. EGFR is a significant epigenetic and metabolic regulator in gliomas [63]. EGFR signaling is activated by mTORC1 and mTORC2, which sense the state of nutrients in the tumor’s microenvironment [64,65]. In EGFR mutated gliomas, the hyperactivation of EGFR leads to epigenetic changes, which promote cancer progression, such as the upregulation of c-Myc and cyclin D1 (CCND1) [66]. This is achieved by translocation of PKM2 into the nucleus and interaction with β-catenin [67]. Upon binding, the resulting complex attaches to the *CCND1* promoter and leads to dissociation of HDAC3, which finally leads to *CCDN1* expression [68]. miR-34c and miR-Let-7a also target c-Myc, a key regulator of glycolysis, as previously mentioned [46,56]. When it comes to the PI3K/Akt pathway that regulates the Warburg effect [69,70], miR-7 targets PI3K [71] and miR-542-3p target Akt [72], whereas miR-503 suppresses PI3K/Akt signaling [73]. Additionally, miR-10a/10b, miR-21, miR-26a, miR-221/222, miR-494-3p, and miR-1908 regulate phosphatase and tensin homolog (PTEN), a GB tumor suppressor and antagonist of PI3K [62,74,75,76,77].

Regarding the liver kinase B1 (LKB1)–AMPK pathway, another major regulator of the Warburg effect [45], miR-451 was detected upregulated in GB, targeting calcium binding protein 39 (CAB39), an LKB1 binding partner that activates AMPK via phosphorylation [78]. Both mTORC2 that influences glycolytic metabolism in GB via FOXO acetylation and c-Myc, are also influenced by miRNAs [46]. More specifically, miR-199a-3p targets mTORC2 [79] but is downregulated in GB, while miR-34a acts on Rictor, an mTORC2 binding partner [80]. miR-let-7a can also directly target PKM2 or target c-Myc and downregulate heterogeneous nuclear ribonucleoprotein A1 (*hnRNPA1*), which in turn, can suppress let-7a [56,81]. hnRNPA1 is responsible for splicing PK into its PKM2 isoform and Max, a c-Myc binding partner into its delta Max isoform. The latter complexes with c-Myc and enhances c-Myc target gene expression [82,83,84]. These interactions between miR-let-7a, PKM2, c-Myc, and hnRNPA1 ensure the upregulation of *PKM2* in GB and, thus, favor glycolysis.

The TP53 target 1 (*TP53TG1*) lncRNA is overexpressed in gliomas, compared with normal brain tissues. It has been shown to promote tumor growth and migration by affecting the expression of genes involved in glucose metabolism at glucose-deprived tumor cells. In more detail, it has been demonstrated to increase the expression of glucose-regulated protein, 78kDa (*GRP78*), and *IDH1* but to decrease PKM2 levels, while its knockdown exhibited the opposite effects [85] (Figure 1).

Lastly, concerning snRNAs and snoRNAs, it has been shown that pseudouridine synthetases may be involved in glioma development, with dyskerin pseudouridine synthase 1 (DKC1) being upregulated in gliomas and correlating with WHO grade [86]. Pseudouridine is a crucial RNA modification that exists in most RNA types [87], playing a major role in their normal function [88]. Even though the metabolic effects of these RNAs are still under elucidation, the impact of pseudouridine synthetases in signaling pathways connected to metabolic alterations, such as HIF-1a, has already been demonstrated [86].

### 2.2. Epigenetic Regulation of Pentose Phosphate Pathway

Tumor cells need to replenish raw materials to support their continuous and rapid proliferation. They can exploit the pentose phosphate pathway (PPP), a glycolysis bypass pathway that produces NADPH and ribose from glucose-6-P, necessary for reductive reactions and nucleotide synthesis, respectively. The transketolase like-1 *(TKTL1)* codes for the respective enzyme participating in the PPP [9]. Promoter hypomethylation of *TKTL1* in cancer cells enhances its expression [89], which is ultimately associated with increased lactate and pyruvate levels that characterize the Warburg effect [90]. TKTL1 also favors the stability and accumulation of HIF1-α, a major molecule for aerobic glycolysis and DNA methylation.

A significant transcriptional activator of key PPP genes, such as *glucose* *6-phosphate dehydrogenase* *(G6PDH)*, *transketolase (TKT)*, and *IDH*, is the nuclear factor erythroid 2-related factor 2 (Nrf2), which ultimately induces cancer cell NADPH and nucleotide synthesis [91]. Physiologically, Nrf2 is constantly degraded depending on kelch like ECH associated protein 1 (KEAP-1) via the ubiquitin-proteasome system [92,93]. Increased promoter methylation downregulates *KEAP-1* expression, activating Nrf2 and subsequently PPP enzymes in glioma cells [94,95] (Figure 1).

### 2.3. Epigenetic Regulation of Gluconeogenesis

Gluconeogenesis converts several non-sugar substrates into glucose and can be modified by DNA methylation. Fructose-1,6-bisphosphatase (FBP), a crucial enzyme of gluconeogenesis exists in two isoforms in humans, namely FBP1 and 2. FBP1 has been suggested to act as a tumor suppressor regulating glucose metabolism by inhibiting aerobic glycolysis and by increasing glucose uptake for macromolecule biosynthesis [96,97]. It is found to be downregulated in many cancer types due to abnormal promoter methylation [9] and is also involved in PKM2 post-translational modifications [98,99,100]. Its loss due to DNA methylation in cancer cells is suggested to contribute to ATP production maintenance [96,101] and OXPHOS inhibition [96], which are key features of the Warburg effect (Figure 1).

### 2.4. Epigenetic Regulation of TCA Cycle

#### 2.4.1. DNA Methylation Effects in the TCA Cycle

**Figure 2:**
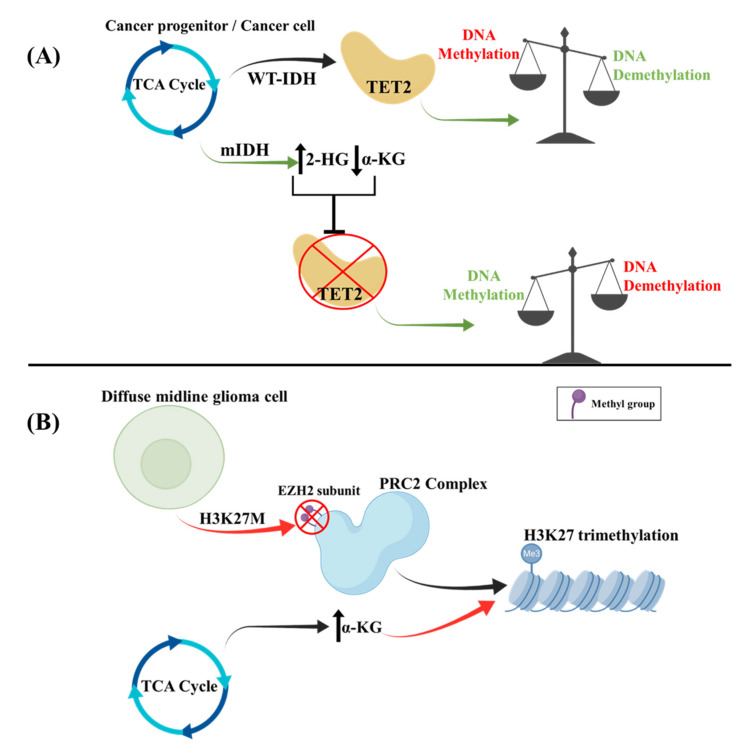
Molecular alterations inducing metabolic reprogramming in gliomas. (**A**) In WT-IDH glioblastoma, α-KG upregulates the action of TET2, which increases the action of DNA demethylases and results in decreased DNA methylation. Conversely, in grade 4 IDH-mutant astrocytomas, mIDH shunts the TCA cycle towards the production of 2-HG instead of α-KG. The decreased levels of α-KG, along with the increased levels of 2-HG, inhibit TET2, which leads to inhibition of DNA demethylases, generating a DNA hypermethylation phenotype. (**B**) In diffuse midline gliomas, H3K27M mutation inhibits the auto-methylation of EZH2, a PRC2 subunit, leading to decreased H3K27me3 levels. Additionally, in these tumors, the TCA cycle is upregulated to generate α-KG which serves as a co-factor for demethylases leading to further reduction in H3K27me3 levels.

Abnormal expression and activity of TCA enzymes may cause mitochondrial dysfunction and promote aerobic glycolysis. PDH is the key regulatory enzyme for pyruvate entry in the TCA cycle, and PDH kinases (PDKs) inhibit its activity in tumors, in favor of aerobic glycolysis [102]. For example, *PDK4* can be upregulated through promoter hypomethylation [103]. Moreover, DNA hypermethylated glioma cell lines are associated with mutations in the gene encoding the TCA cycle enzyme IDH1/2. During the TCA cycle, mutant IDH modulates the metabolic pathway to generate the oncometabolite (*R*)-2-hydroxyglutarate (2-HG) instead of the physiologically produced α-ketoglutarate (α-KG) [104]. This metabolic alteration disrupts the function of the α-ketoglutarate-dependent ten-eleven translocation 2 (TET2) enzyme, which normally catalyzes the production of 5-hydroxymethylcytosine from 5-methylcytosine in the presence of α-KG, iron, and oxygen, a process that subsequently favors DNA demethylation (Figure 2). Mutations of α-ketoglutarate thus prevent this reaction and result in a GB hypermethylated DNA state [104]. In regard to the tumor microenvironment of cancer cells, 2-HG has been shown to cause HIF-1a destabilization, ultimately leading to enhancement of oxidative phosphorylation, decreased Th17 T-cell polarization, and increased regulatory T-cell activity [105].

**Figure 3:**
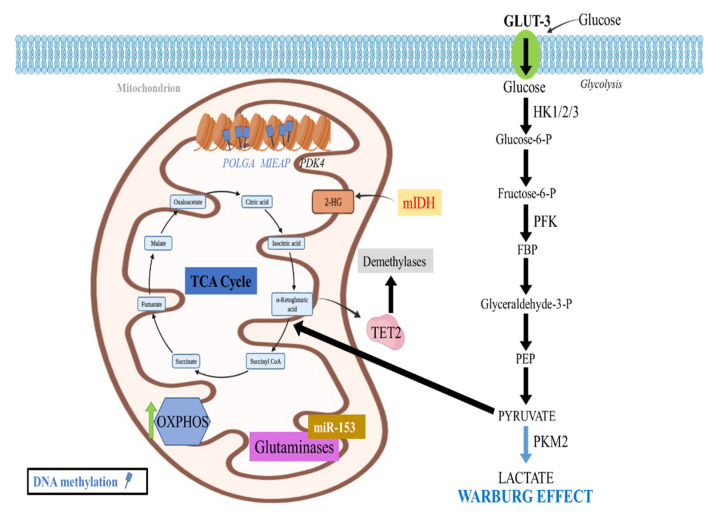
Crosstalk of epigenetic changes and mitochondrial metabolism in GB. High -grade GB mitochondrial DNA is heavily methylated, especially at the POLGA gene, resulting in increased glycolysis instead of OXPHOS to sustain high proliferation rates. Mieap downregulation through promoter methylation further contributes to mitochondrial dysfunction. PDK4 is upregulated through promoter hypomethylation in favor of aerobic glycolysis. Glioma DNA hypermethylation is also associated with IDH1/2 mutations, which generate the 2-HG oncometabolite instead of the α-KG. This decreases the activity of TET2 enzyme and its downstream demethylases, promoting a hypermethylation phenotype. miRNA-153 may act on glutaminases to decrease glutamine metabolism, leading to lactate build up, nucleotide production, and tumor resistance to treatment.

Other mutated TCA cycle enzymes include fumarate hydratase [106] and succinate dehydrogenase, resulting in succinate and fumarate accumulation, which inhibit TET enzymes as well as the degradation of HIF, promoting the Warburg effect and enhancing the methylation of the entire genome, respectively [107,108] (Figure 3).

#### 2.4.2. Histone Methylation Effects in the TCA Cycle

In pediatric diffuse gliomas and more specifically in diffuse midline gliomas, the H3K27M mutation (which involves the replacement of lysine by methionine at site 27 of histones H3-H3F3A and HIST1H3B/C) is responsible for the upregulation of TCA cycle metabolism in parallel with increased expression of hallmark metabolic enzymes, such as HK2, GLUD1, and IDH1 [109]. Furthermore, α-KG, an intermediate of the TCA cycle, serves as a co-factor for demethylases, which keep the H3K27me3 levels low. This interaction works to counteract the inhibitory effect of H3K27me3 on gene expression to keep the chromatin in a more “open” and accessible state. The inhibition of α-KG formation leads to a disruption of this interaction and promotes a “closed” chromatin state as well as decreased tumor cell proliferation, which correlate with elevation of the H3K27me3 levels (Figure 1). Lastly, mutated IDH1/2 can decrease α-KG levels. Another mechanism by which H3K27M leads to reduced H3K27me3 levels involves its interaction with PRC2. H3K27M is able to bind the EZH2 component of the PRC2 complex, resulting in its inactivation through an unknown mechanism, probably through inhibition of EZH2 automethylation [110,111].

In addition to the above, diffuse midline gliomas with H3K27M exhibit increased glycolysis and glutaminolysis, which are also correlated with the upregulation of the aforementioned enzymes HK2, GLUD1, and IDH1 [109]. Enhanced glycolysis along with glutaminolysis further induce an elevation of the α-KG/succinate ratio, promoting the activation of genes related to cell proliferation (Figure 3).

### 2.5. Epigenetic Regulation of Oxidative Phosphorylation (OXPHOS)

#### 2.5.1. DNA Methylation Effects in OXPHOS

Apart from glycolysis, DNA methylation also influences OXPHOS and mitochondrial function. The mitochondrial DNA (mtDNA) is essential for energy production through oxidative phosphorylation, coding for 13 ETC subunits. Pluripotent and tumor cells, including high-grade GB cell lines appear to be heavily DNA methylated especially in exon 2 of DNA polymerase gamma, catalytic subunit (*POLGA*) and are characterized by lower mtDNA copies [104], therefore mainly relying on glycolysis instead of OXPHOS in order to sustain their high proliferation rates [112]. Several compounds, however, that may cause DNA demethylation through different mechanisms, such as vitamin C (VitC) or the drug 5-azacytidine (5-Aza) [104], have been shown to reverse this hypermethylated state and to lead to increased mtDNA copy number, rendering the mitochondrial genome more accessible to replication. Therefore, the differentiation of these cells is then promoted and the increased requirements for OXPHOS-produced ATP are fulfilled.

Mitochondrial dysfunction and ROS accumulation have been often observed in tumor cell lines due to the accumulation of altered mitochondria. This has been attributed to promoter methylation and thus downregulation of mitochondria-eating protein (*Mieap*), which serves as a mitochondrial quality control protein. Mieap normally eliminates oxidized proteins [113,114] by increasing the entrance of lysosomal proteins into the mitochondria without damaging the mitochondrial membrane. Lysosomes can then absorb oxidized proteins and simultaneously increase ATP synthesis while decreasing ROS generation [115] (Figure 3).

#### 2.5.2. Histone Methylation Effects in OXPHOS

As mentioned above, MLL1 of glioma stem cells inhibits HIF transcript expression, preventing the transcription of its target genes, which include glycolysis-related genes [38]. Specifically, *Gluts, LHDA, PFK*, and *HKs* are not expressed and mitochondrial oxygen consumption as well as OXPHOS are increased [39].

#### 2.5.3. Noncoding RNAs Effects in OXPHOS

GBs rely on aerobic glycolysis for energy production, but they are also characterized by mitochondrial dysfunction. ATP synthase F1 subunit alpha (*ATP5A1*) and ATP synthase F1 subunit beta (*ATP5B)*, which are components of the ETC, were detected upregulated in GB, especially in high microvascular proliferation states [116]. Of note, Let-7f and miRs-16, -23, -100, and -101 were able to target and downregulate *ATP5A1* and *ATP5B*, possibly reducing tumor growth and microvascular proliferation.

It is therefore evident that a complex interplay between epigenetic and metabolic pathways regulates the growth and proliferation of neoplastic cells in these tumors (Table 1).

### 2.6. Epigenetic Regulation of Lipid Metabolism

#### microRNA Effects in Lipid Metabolism

GB is characterized by changes in normal lipid metabolism. Examples involve the increased expression of SREBP cleavage-activating protein (*SCAP*) and transcription factor sterol regulatory element binding protein 1 (*SREBP-1*), which participates in cholesterol synthesis [120]. The signaling pathway of EGFR enhances miR-29 expression in GB by upregulating *SCAP* and *SREBP-1*. In turn, SREBP-1 induces miR-29, which ultimately inhibits the expression of *SCAP* and *SREBP-1* through interactions with their 3’ untranslated regions (UTRs) suppressing lipid synthesis and GB cell growth. This effect can be reversed with the addition of N-terminal SREBP-1 or fatty acids [117,118]. This miR-29-SCAP/SREBP-1 feedback loop can thereby modulate EGFR signaling-associated GB growth by altering cholesterol synthesis [117,118] (Figure 1).

### 2.7. Epigenetic Regulation of Glutamine Metabolism

Increased rates of aerobic glycolysis in gliomas enhance the demand for substrate replenishment mechanisms. One solution to this problem is glutamine, which serves as a carbon source for the TCA cycle but may also be redirected through the malate shuttle to offer additional energy, allowing accumulation of lactate [121]. Glutamine is also essential for nucleotide production and enhances the function of the glutathione (GSH) redox system, offering gliomas resistance to chemo- and radiotherapy. Interestingly, epigenetic mechanisms also seem to influence glutamine metabolism. One example is miRNA-153, which acts on glutaminases to decrease metabolism of glutamine in GB [119] (Figure 3).

Of note, α-KG production through glutaminolysis participates in the alternative macrophage activation (M2), which restrains proinflammatory responses, as well as in fatty acid oxidation promotion and M2 gene epigenetic reprogramming. In more detail, a higher α-KG/succinate ratio favors the M2 phenotype, while a lower ratio favors the proinflammatory phenotype (M1) of classically activated macrophages, therefore influencing the immune response in GB [122].

## 3. Therapeutic Targeting Options

The manipulation of the abovementioned epigenetic and metabolic pathways has been suggested as a promising treatment option for gliomas either as monotherapy or in combination with the current standard treatment regimen: temozolomide and radiation therapy. DNA methylation primarily regulates the Warburg effect, which is important for tumor development. Drugs that help overcome OXPHOS dysregulation or inhibit glycolysis are therefore being proposed as new strategies for cancer treatment. Examples include the glycolysis inhibitor 3-bromopyruvate (3-BP), which targets HK-2 and the mitochondrial ATP synthasome [123]; the glucose analog 2-deoxy-d-glucose (2-DG), which depletes tumor cell energy reserves and elicits antitumor effects [124], as well as dichloroacetic acid (DCA), which can minimize the Warburg effect through PDK1 inhibition, thus shifting tumor cells towards OXPHOS for glucose metabolism [125].

Another interesting approach in the treatment of GBs could be the manipulation of ROS through changes in DNA methylation. Already tested in breast and ovarian cancer, decitabine works as a DNA methylation inhibitor that upregulates ROS production, subsequently increasing the sensitivity to Poly (ADP-ribose) polymerase (PARP) inhibitors [126]. Another example is the use of the live-attenuated measles virus vaccine in ovarian cancer cells, which causes ROS upregulation. This in turn leads to DNA methylation and *E-cadherin* gene silencing, promoting loss of intercellular contact and cancer cell apoptosis [127]. Similarly, RRx-001, a hypoxia-selective epigenetic agent increased ROS and nitrogen production while it inhibited DNMTs and DNA methylation, inducing apoptosis in drug resistant multiple myeloma lines [128]. This compound is being used in a phase I clinical trial combining RRx-001 with the standard radiation and temozolomide treatment in newly diagnosed GB and anaplastic gliomas (NCT02871843).

In regard to histone deacetylation, pan-HDAC inhibition and synergistic blockade of glycolysis can also lead to GB cell apoptosis [129]. Nicotinamide (Nico), a sirtuin (class III NAD +-dependent HDAC) inhibitor; SAHA (vorinostat), a pan-HDAC inhibitor; and tubastatin A (TUBA), an HDAC inhibitor, can act on mitochondrial metabolism, glycolysis, as well as fatty acid synthesis in GB [130]. Using niacin (a NAD+ prodrome compound) a phase I/II clinical trial has been initiated to evaluate whether the addition of niacin along with the standard temozolomide and radiation therapy treatment will provide a significant effect in IDH-mutant astrocytoma grade 4 (NCT04677049). Another phase I clinical trial is investigating the combination of vorinostat and temsirolimus with or without radiotherapy in patients with H3K27M diffuse midline glioma (NCT02420613). The HDAC inhibitor 4-phenylbutyrate (4-PB), was shown to suppress the mRNA expression of GAPDH in gliomas, decreasing energy consumption and cell proliferation [131]. Histone deacetylation may thus be a promising strategy in the treatment against GB [9].

Moreover, HDAC inhibitors, romidepsin (FK228) in particular (a HDAC1/2 inhibitor that can also act on non-histone targets), are able to modulate various metabolic signaling pathways targeting tumor-specific transcription factors in different solid cancers including glioblastoma. To date, several lines of evidence support the testing of novel combinatorial therapeutic strategies based on the combination of drugs commonly used in clinical practice and HDAC inhibitors such as FK228 to improve therapeutic efficacy in GB patients [132,133].

Regarding pediatric diffuse midline gliomas with H3K27M mutation, several trials have tackled a variety of therapeutic approaches to increase patients’ survival. In an attempt to inhibit α-KG-producing enzymes, Chung et al. used JHU-083 (glutamine antagonist DON analog) and WT-DH1i13 (micro molecules, which covalently suppress WT-IDH1) [109]. Both agents exhibit high BBB permeability, and their combination was shown to induce maximal therapeutic response. Moreover, the drug combination was demonstrated to prolong the overall survival in a mouse model bearing diffuse midline glioma with H3K27M mutation [109].

Another potential treatment option employs panobinostat, a pan-HDAC inhibitor that has been previously used to treat multiple myeloma along several other cancer types [134]. Panobinostat exhibits both in vitro and in vivo efficacy in treating diffuse midline gliomas with H3K27M mutation. Its therapeutic effect increases when combined with GSKJ4, a JMJD3 (histone demethylase jumonji D3) demethylase inhibitor [135]. The study by Souweidane et al. demonstrated the beneficial effects of convection-enhanced mode of panobinostat delivery in avoiding the toxicity observed with its use [136]. Moreover, a completed phase I/II trial has used the panobinostat nanoparticle formulation MTX110 in patients with diffuse midline gliomas with H3K27M and may potentially shed more light in both the use of panobinostat as well as the nanoparticle formulation for effective patient delivery (NCT03566199). Two phase I clinical trials are also evaluating the use of panobinostat in gliomas. the first one investigating its effects in patients with H3K27M diffuse midline gliomas (NCT02717455) and the other exploring the use of non-invasive focused ultrasound to safely trans pass the BBB, thus aiming to increase the concentration of panobinostat in patients with H3K27M diffuse midline gliomas, treated with oral panobinostat (NCT04804709).

As previously mentioned, the metabolic changes coupled with the epigenetic changes in diffuse midline gliomas bearing H3K27M lead to a global H3K27 hypomethylation phenotype that can be targeted to treat patients with this tumor. Therefore, other pharmacologic targeting options focus either on enhancing the activity of methyltransferases or reducing the activity of H3K27 demethylases or a combination of the two. GSKJ4, a previously mentioned JMJD3 inhibitor was shown to increase the levels of H3K27me2 and H3K27me3 specifically in glioma cells harboring the H3K27M [137], leading to decreased colony formation, cell viability, as well as increased apoptosis. The administration of GSKJ4 also resulted in increased median survival and enhanced radiosensitivity by inhibiting the homologous recombination DNA double-strand break repair pathway (HR DNA DSB). The inhibition of HR DNA DSB was noted only in cells harboring the H3FEA mutant K27M. The major drawback of using GSKJ4 relies on the fact that it is a prodrug that rapidly converts to the active compound (GSK-J1) that, unfortunately, does not readily cross the cell membrane [137]. This also underlines the importance of research in new ways of delivering drugs to these types of tumors.

Furthermore, it has been noted that these tumor cells harbor increased levels of H2K37ac, which co-localizes with BrD4 in their nucleosomes. Using JQ1 (a BET bromodomain and extra-terminal domain inhibitor) to inhibit BrD4 was shown to lead to a neuron-like cell phenotype and decreased proliferation. In parallel, markers of differentiation such as CDKN1A, TUBB3, and MAP2 were upregulated [110]. The use of JQ1 was also able to prolong survival in vivo and to decrease tumor size after 10 days of treatment. Moreover, H3K27ac in mice treated with JQ1 was also significantly decreased. Using another BET inhibitor, I-BET151, similar results were noted with markedly decreased tumor growth in vivo. A combination of Panobinostat with JQ1 or THZ1 (a CDK7 inhibitor) demonstrated increased antitumor effects, with lower tumor cell proliferation and increased apoptosis [138].

Lastly, in diffuse midline gliomas harboring H3K27M, the increased H3K27ac has also been associated with endogenous retroviruses (ERVs) [139]. ERV silencing is normally achieved by the inhibitory effects of H3K9me3 as well as H3K27me3 [140]. Panobinostat and 5-azacytidine have been shown to derepress ERVs by increasing H3K27ac and by decreasing H3K27me3 levels, respectively [139]. 5-azacytidine has the ability to incorporate into DNA and to decrease DNA methylation, which can also promote ERV derepression [141]. A phase II clinical trial using 5-azacytidine in recurrent gliomas with mIDH1/2 is also underway (NCT03666559). Furthermore, ERV derepression using low doses of 5-azacytidine combined with panobinostat can elicit a toxic effect on glioma cells, in turn, triggering viral mimicry (a state in which innate cellular responses similar to those of a cell infected by a virus are activated) and promoting interferon production, thus activating immune cells and cell death [139,142]. Interferon production is upregulated by DNA pattern receptors, such as RIG-1 and MDA5, which recognize the double stranded RNA produced by derepressed ERVs [143].

Altogether, this experimental evidence demonstrates how the effect of drugs already used in cancer therapy can be combined with current treatments to achieve a more favorable result (Table 2). Further exploration of the mechanisms that lead to cancer development and novel therapy combinations that target all the dysfunctional pathways hold great potential for providing a better therapeutic outcome.

## 4. Conclusions

Taken altogether, it is evident that malignant cells exhibit an altered metabolic program, which is largely attributed to alterations in epigenetic modifications. GBs are no exception, with changes in metabolic regulation being associated with aggressive behavior and poor prognosis. Metabolic alterations are also partly responsible for the heterogeneity that characterizes GBs, since different cell subsets exert distinct metabolic phenotypes. Moreover, it contributes to their plasticity, since GB cells can alter their metabolism depending on the endogenous and exogenous stimuli. One of the hallmark metabolic alterations in GBs is the Warburg effect, where malignant cells shunt their glucose stores towards aerobic glycolysis and the pentose phosphate pathway, thus reducing its use in oxidative phosphorylation.

The influence of epigenetics is pivotal in affecting the metabolic pathways taking place in GBs. In this scope, epigenetic modifications can regulate glycolysis, oxidative phosphorylation, the TCA cycle, lipid, and glutamine metabolism, mainly favoring glycolysis over OXPHOS. Many epigenetic changes can exert contradicting effects, depending on the type of epigenetic modification as well as the genomic region where it takes place. The full network of genes, together with their epigenetic modifications in which altered expression affects the metabolic pathways, compose the metabolic phenotype of each cell.

Unfortunately, despite the extensive research on the field of metabolic changes in cancer and epigenetics, the pathophysiology of these alterations is still under investigation and their targeting options are limited. This is attributed to the high complexity of the interplay between the metabolic and epigenetic networks and to the limitation that current studies have been mainly conducted in vitro and only a few times in in vivo models. Targeting epigenetic modifications also poses a challenge, since drug specificity for cancer cells is low, making the response of normal cells potentially harmful. Lastly, epigenetic mechanisms are not the only drivers of metabolic changes observed in cancer, making their selective targeting even more challenging.

The standard therapy for GB remains, to date, the combination of temozolomide with radiation therapy. However, GB have proven to be resilient to therapy and their high rate of recurrence creates a therapeutic dead-end. Many studies indicate the beneficial combination of standard treatment with novel regulators of the epigenome or metabolism; however, their results are still exploratory and do not provide a clear therapeutic advantage for glioma patients.

More research studies are therefore required to fully elucidate the entirety of the epigenetic network and the extent to which it affects GB cell metabolism. Further studies using in vivo models are highly demanded along with an extensive analysis of the epigenome and metabolome to detect patients who will respond more readily to their targeting. Lastly, the development of more specific targeting methods and future research into tumor-selective delivery drug systems will be of paramount importance to apply this knowledge in clinical practice.

## Figures and Tables

**Table 1:** Epigenetic changes and associated metabolic genes, enzymes, and pathways.

Enzyme/ Molecule	Classification	Effect on Metabolism	Key Metabolic Genes/ Enzymes/Pathways Involved	Reference
G9	HMT	Inhibits glycolysis	HIF-1, *SLC6A3*, *PTGS1*, *NDNF*, *Linc01132*	[37]
MLL1	HMT	Inhibits glycolysis, increases OXPHOS	*Gluts*, *HKs*, *LHDA*, *PFK*	[38,39]
EZH2	HMT	Favors glycolysis	*EAF2*	[36]
LSD1/HDM1A	HDM	Inhibits glycolysis	p53	[31]
miR-215/KDM1B	microRNA and HDM	Favor glycolysis	HIF2α, *Ndrg1*, *ADM*, *NDUFA4L2*, *Glut1*, *Glut3*	[42]
PCAF/KAT2B	HAT	Favors glycolysis	Akt1	[43]
KAT6A	HAT	Favors glycolysis	PI3K/Akt, *Glut1*, *PFK1*, *HK1/2/3*	[44]
HDAC 4/5/7	HDAC	Favors glycolysis	c-Myc, *ENO1*, *Glut1*, *LDH-A, SHMT*	[46]
SIRT6	HDAC	Inhibits glycolysis	*HK2*	[48]
miR-143	microRNA	Inhibits glycolysis	HK2	[54]
miR-let-7a	microRNA	Favors glycolysis	c-Myc, PKM2	[55,56]
miR-29	microRNA	Decreases lipid synthesis	*SCAP*, *SREBP-1*	[117,118]
miR-153	microRNA	Decreases glutamine metabolism	glutaminases	[119]

**Table 2:** Drugs targeting the epigenetic or metabolic interplay in gliomas.

Drug Name	Mechanism of Action	Type of Study	Reference
**Glycolysis inhibitors**			
3-Bromopyruvate (3-BP)	Inhibits glycolysis by targeting HK-2 and the mitochondrial ATP synthasome	Preclinical studies in Various cancers	[123]
2-deoxy-D-glucose (2-DG)	Glucose analog that depletes tumor cell energy, eliciting antitumor effects	Phase 1 clinical trials in GB	[124]
Dichloroacetic acid (DCA)	PDK1 inhibitor that minimizes the Warburg effect and shifts tumor cells towards OXPHOS	Preclinical trials in ovarian cancer	[125]
**DNA methylation** **inhibitors**			
5-azacitidine	Gets incorporated into the DNA and its residues inhibit DNA methylation	Phase II clinical trial in GB	[141]
**ROS manipulators**			
Decitabine	Upregulates ROS production, increases sensitivity to PARP Inhibitors	Preclinical studies in Ovarian and breast cancer	[126]
Live-attenuated measles vaccine	Upregulates ROS, causing DNA methylation and *E-cadherin* silencing, leading to intercellular loss of contact and cancer cell apoptosis	Preclinical studies in Breast cancer	[127]
RRx-001	Increases ROS and nitrogen production, inhibits DNA methylation, leading to apoptosis	Phase I clinical trial in gliomas	[128]
**Deacetylase inhibitors**			
Nicotinamide	Sirtuin (class III NAD^+^-dependent HDAC) inhibitor that can affect mitochondrial metabolism, glycolysis, and fatty acid synthesis in GB	Phase I/II clinical trial	[130]
Vorinostat (SAHA)	Pan-HDAC inhibitor that can affect mitochondrial metabolism, glycolysis, and fatty acid synthesis in GB	Phase I clinical trial	[130]
Tubastatin A	Selective HDAC inhibitor that can affect mitochondrial metabolism, glycolysis, and fatty acid synthesis in GB	Preclinical studies in GB	[130]
4-phenylbutyrate	Selective HDAC inhibitor that suppresses GAPDH mRNA expression, decreasing energy consumption and cell proliferation	Preclinical studies in gliomas	[131]
Panobinostat	Pan-HDAC inhibitor, more effective when combined with low dose 5-azacytidine in derepressing ERVs and activating the immune system leading to glioma cell death	Phase II clinical trial	[135]
**α-KG- producing** **enzyme inhibitors**			
JHU-083 and WT-DH1i13	Glutamine antagonist and WT-IDH1 antagonist prolonged survival in diffuse midline gliomas with H3K27M	Preclinical studies in diffuse intrinsic pontine gliomas	[109]
**Lysine demethylase inhibitor**			
GSKJ4	JMJD3 lysine specific demethylase inhibitor increases H3K27me2 and H3K27me3 levels in glioma cells harboring the H3K27M, decreasing cell viability and colony formation	Preclinical studies in diffuse intrinsic pontine gliomas	[137]
**BET bromodomain** **and extra- terminal** **domain inhibitor**			
JQ1	BET bromodomain and extra-terminal domain inhibitor leading to a more differentiated cellular phenotype and decreased proliferation	Preclinical studies in diffuse intrinsic pontine gliomas	[110]
